# Structural basis for polymerase η–promoted resistance to the anticancer nucleoside analog cytarabine

**DOI:** 10.1038/s41598-018-30796-w

**Published:** 2018-08-23

**Authors:** Olga Rechkoblit, Jayati Roy Choudhury, Angeliki Buku, Louise Prakash, Satya Prakash, Aneel K. Aggarwal

**Affiliations:** 1 Department of Pharmacological Sciences, Icahn School of Medicine at Mount Sinai, Box 1677, 1425 Madison Avenue, New York, NY 10029 USA; 2 Department of Biochemistry and Molecular Biology, University of Texas Medical Branch, 301 University Boulevard, Galveston, TX 77755–1061 USA

## Abstract

Cytarabine (AraC) is an essential chemotherapeutic for acute myeloid leukemia (AML) and resistance to this drug is a major cause of treatment failure. AraC is a nucleoside analog that differs from 2′-deoxycytidine only by the presence of an additional hydroxyl group at the C2′ position of the 2′-deoxyribose. The active form of the drug AraC 5′-triphosphate (AraCTP) is utilized by human replicative DNA polymerases to insert AraC at the 3′ terminus of a growing DNA chain. This impedes further primer extension and is a primary basis for the drug action. The Y-family translesion synthesis (TLS) DNA polymerase η (Polη) counteracts this barrier to DNA replication by efficient extension from AraC-terminated primers. Here, we provide high-resolution structures of human Polη with AraC incorporated at the 3′-primer terminus. We show that Polη can accommodate AraC at different stages of the catalytic cycle, and that it can manipulate the conformation of the AraC sugar via specific hydrogen bonding and stacking interactions. Taken together, the structures provide a basis for the ability of Polη to extend DNA synthesis from AraC terminated primers.

## Introduction

**Figure 1:**
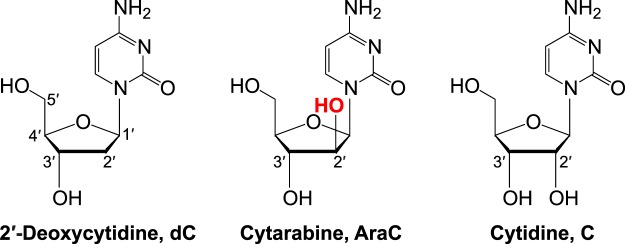
Deoxycytidine, cytarabine, and cytidine nucleosides. Chemical structures of 2′-deoxycytidine (dC, 1-β-D-2′-deoxy-ribofuranosylcytosine), cytarabine (AraC, 1-β-D-arabinofuranosylcytosine), and cytidine (C, 1-β-D-ribofuranosylcytosine). The 2′-OH group in arabinose and ribose sugar moieties points in opposite directions.

Cytarabine (1-β-D-arabinofuranosylcytosine, AraC) has been the mainstay therapy for acute myeloid leukemia (AML) for over 40 years^1–3^. Resistance to cytarabine is a major cause of treatment failure and only ~27% of adult patients survive more than 5 years after the initial diagnosis^4^. AraC is a nucleoside analog that differs from 2′-deoxycytidine only by the presence of an additional hydroxyl group at the C2′ position of the 2′-deoxyribose (Fig. 1). This 2′-OH of the arabinose sugar moiety points in an opposite direction to that of the 2′-OH of the ribose sugar in ribonucleotides (Fig. 1).

AraC is a prodrug, which after entering the cell is phosphorylated by deoxycytidine and pyrimidine kinases to its active form, AraC 5′-triphosphate (AraCTP)^5^. AraC kills cells undergoing DNA synthesis in the S-phase of the cell cycle and its chemotherapeutic action is primarily associated with DNA fragmentation and chain termination^5^. Concordantly, high-fidelity replicative DNA polymerases α, δ and ε that replicate the bulk of genomic DNA in eukaryotes^6,7^ can proficiently insert AraC at the 3′ terminus of a growing DNA chain but the subsequent extension reaction is markedly impeded^8–11^. Nevertheless, a substantial fraction of AraC-terminated primers do get extended^12^, and thereby reducing the effectiveness of the chemotherapy. This has been attributed to specialized, lower fidelity translesion DNA synthesis (TLS) polymerases, which allow for the continuity of the replication fork by allowing replication through modified DNA bases that impede the high-fidelity replicative polymerase^7,13^. TLS polymerase η (Polη) stands out in that human cells deficient in Polη are ~3-fold more sensitive to AraC than wild-type cells and the polymerase can efficiently extend AraC-terminated primers *in vitro*^14^.

To see how Polη can extend AraC-terminated primers we present here high-resolution crystal structures of human Polη in binary and ternary complexes with AraC. We show that Polη can accommodate AraC at different stages of the catalytic cycle, and that it can manipulate the conformation of the AraC sugar via specific hydrogen bonding and stacking interactions. Taken together, the structures provide an unexpected basis for the ability of Polη to extend DNA synthesis from AraC terminated primers.

## Results

### Biochemical Analysis

**Figure 2:**
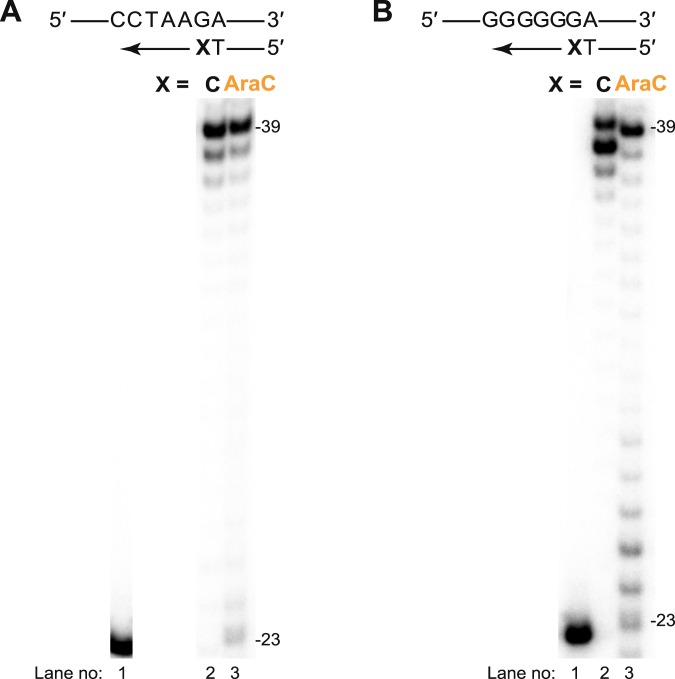
Polη-catalyzed extension from AraC 3′-terminated primers. (**A**) Extension of ^32^P 5′-end-labeled 23-nt primer with the unmodified C or AraC residues at the 3′-end bound to the 5′-…CCTAA**G**A…-3′ 39-nt template in the presence of dATP, dGTP, and dTTP. Lane 1, unmodified DNA substrate; Lane 2, extension from the unmodified C; Lane 3, extension from AraC. Lanes 1, 2 and 3 were run on the same gel, but lane 1 was cropped from a different part of the gel. Thus, lane 1 is separated with white space from lanes 2 and 3. The original gel is shown in Supplementary Fig. 1. (**B**) Extension with the 5′-…GGGGG**G**A…-3′ 39-nt template. Lane 1, unmodified DNA substrate; Lane 2, extension from the unmodified C in the presence of dATP, dGTP, dTTP and dCTP; Lane 3, extension from the AraC in the presence of dATP, dGTP, dTTP and AraCTP. Lanes 1–3 were run together on the same gel. The original gel is shown in Supplementary Fig. 2. All extension reactions were conducted at 37 °C for 10 min.

We first carried out biochemical analysis to compare the ability of human Polη to extend DNA synthesis from an unmodified C and AraC 3′-terminated DNA primers. As shown in Fig. 2A (lane 2), with a 5′-…CCTAA**G**A…-3′ DNA template (5′-GTCTAATACTTCCTAA**G**ATGCCTACACTGGAGTACCGGA-3′), elongation from unmodified C paired with the cognate template **G** base results in nearly complete extension to the full products in 10 min of the reaction time. A slightly lesser amount of the extension products is observed with the AraC-terminated primer (Fig. 2A, lane 3). Likewise, with a 5′-…GGGGG**G**A…-3′ DNA template that contains six consecutive G residues, extension from the 3′-terminal unmodified C or AraC opposite the first **G** of the template strand results in the nearly complete products (Fig. 2B, lanes 2 and 3). We conducted primer elongation on the G-repeat template in the presence of all four dNTPs with the unmodified primer, whereas, with the AraC-terminated primer, we used AraCTP, dATP, dGTP and dTTP nucleotides. Thus, the efficient formation of the fully extended products (Fig. 2B, lane 3) suggest that Polη can not only extend from a single AraC residue but also can consequently incorporate and extend from it to create a run of AraC bases. Overall, our results demonstrate that Polη is proficient in extension from AraC-terminated primers in various sequence contexts and are in accord with previous observations^14^.

### Structure determination

We crystalized the human Polη catalytic core (residues 1 to 432) with a DNA 12-nucleotide (nt) template (5′-CATGACAGTGCT-3′)/8-nt primer (5′-AGCACTGT-3′) and AraCTP. However, when we solved the structure and refined it to 1.83 Å resolution, we discovered that despite the suboptimal reaction conditions in the crystallization drop (low pH 6.0 and presence of Ca^2+^ ions) Polη had covalently added the AraC residue to the 3′ end of the primer strand. This represents a “post-insertion” binary complex in which polymerase has not yet translocated to the next templating position. To capture the polymerase in the act of inserting dNTP from an AraC terminated primer, we first incubated the polymerase with a 12-nt template (5′-CATTGCAGTGCT-3′)/7-nt primer (5′-AGCACTG-3′) and AraCTP at pH 8.0 and in the presence of Mg^2+^ to incorporate AraC at the primer terminus, followed by removal of unincorporated AraCTP, and then addition of nonreactive dATP analog dAMPNPP (2′-deoxyadenosine-5′-[(α,β)-imido]triphosphate) to the crystallization mix. This represents a ternary complex in which AraC is incorporated at 3′ end of the primer and dAMPNPP is the incoming nucleotide. The structure was refined to high 1.75 Å resolution and provides atomic details on the conformation of AraC and its interactions with the polymerase. The crystal data, data collection statistics, and refinement statistics for both complexes are summarized in Table 1.

**Table 1:** X-ray data collection and refinement statistics.

	AraC post-insertion binary complex	AraC extension ternary complex
**Data collection**		
Space group	P6_1_	P6_1_
Cell dimensions:		
*a*, *b*, *c* (Å)	98.6 98.6 81.5	98.8 98.8 81.8
α, β, γ (°)	90.0, 90.0, 120.0	90.0, 90.0, 120.0
Resolution range (Å)^a^	45.0–1.83 (1.86–1.83)	85.6–1.75 (1.78–1.75)
*R*_merge_ (%)	7.4 (61.2)	8.5 (26.5)
*I*/σ*I*	31.4 (2.3)	17.4 (1.1)
Completeness (%)	99.7 (95.7)	100 (100)
Redundancy	12.0 (7.3)	16.6 (16.4)
CC_1/2_ (%)	100 (80.7)	100 (60.5)
**Refinement**		
Resolution range (Å)	42.7–1.83	59.1–1.75
No. reflections	39,435	45,739
*R*_work_/*R*_free_	16.0/19.6	16.8/19.5
No. atoms		
Protein	3,411	3,371
DNA	431	438
Ligand (dAMPNPP)	0	1
Ligand (other)	6	2
Ion (Mg^2+^)	0	2
Water	436	374
*B*-factors		
Protein	26.5	42.8
DNA	30.1	44.8
Ligand (dAMPNPP)	−	29.1
Ligand (other)	16.3	45.6
Ion (Mg^2+^)	−	35.0
Water	33.2	43.6
R.m.s. deviations		
Bond length (Å)	0.006	0.010
Bond angles (°)	0.83	1.04

^a^Values in parentheses are for highest-resolution shell.

### Overall Arrangement

**Figure 3:**
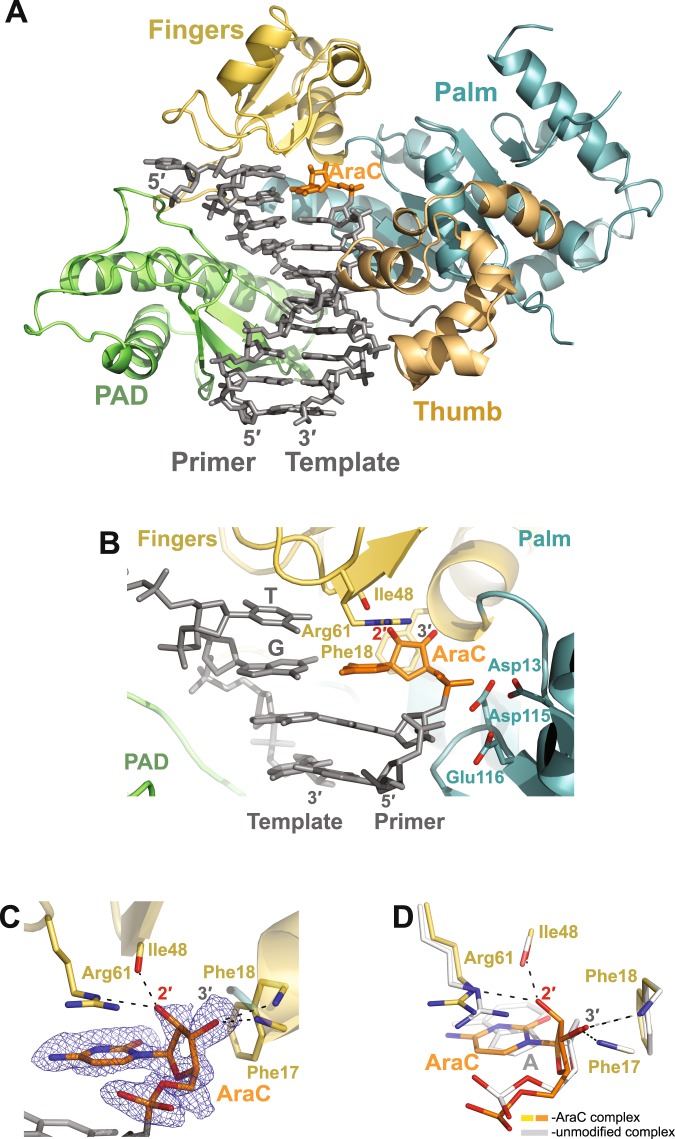
The Polη AraC post-insertion binary complex. (**A**) Overall structure of the complex; the palm, fingers, thumb and PAD domains are shown in cartoon representation in cyan, yellow, light orange, and green, respectively. The DNA template–primer duplex is shown in gray sticks with the 3′-terminal primer AraC residue in orange. (**B**) A close-up view of the AraC in the active site of Polη. Asp13, Asp115, and Glu116 are the catalytic residues. (**C**) A simulated annealing Fo − Fc omit map (contoured at 3.0σ-level at 1.83 Å resolution and colored in blue) showing the clear electron density for the entire AraC residue. The sugar moiety of the AraC is stacked against the steric gate residue Phe18 and its 2′-OH group points away from it. (**D**) Superposition of the AraC and the unmodified (PDB ID: 4J9P)^36^ post-insertion binary complexes. The unmodified complex is in white and has an unmodified A residue at the 3′-end of the primer strand; the structures are superimposed by the palm and fingers domains of the polymerase. Both, the AraC and the unmodified A sugar moieties have C3′-*endo* conformations.

**Figure 4:**
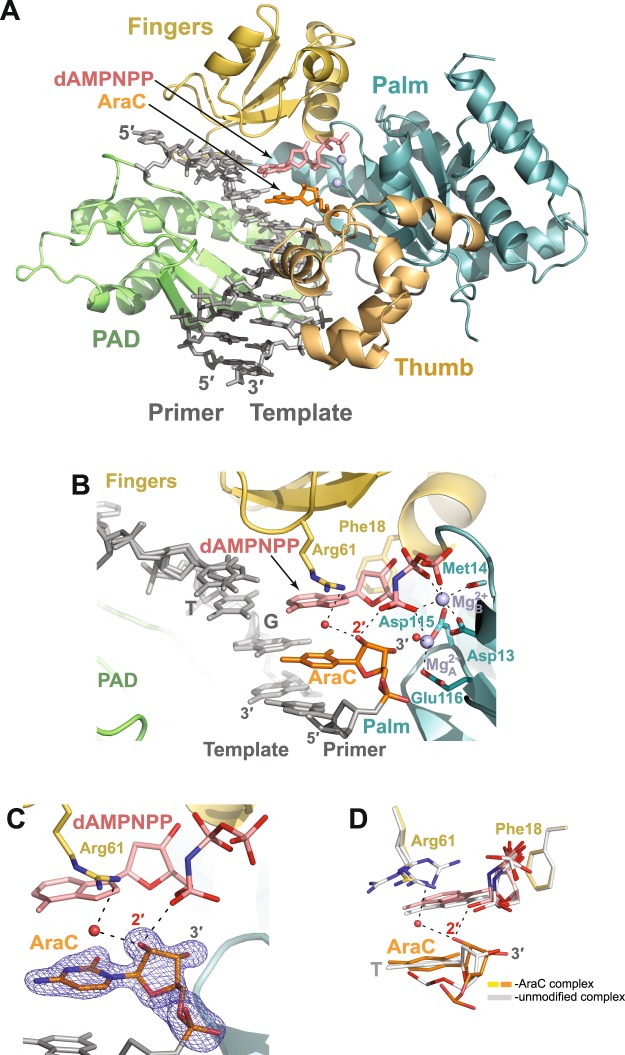
The Polη AraC extension ternary complex. (**A**) Overall structure of the complex. The incoming dAMPNPP residue is in red. The Mg^2+^ ions A and B are represented as light blue spheres. The other colors are as described in Fig. 3. (**B**) The active site of the AraC extension ternary complex. Asp13, Asp115, and Glu116 are the catalytic residues. The water molecules coordinated by the 2′-OH of the AraC and by the Mg^2+^ B-ion are shown as red spheres. (**C**) A simulated annealing Fo–Fc omit map (contoured at 3.0σ-level at 1.75 Å resolution and colored in blue) showing the clear electron density for the entire AraC residue. (**D**) Superposition of the AraC and the unmodified (PDB ID: 3MR2)^27^ ternary complexes. The unmodified complex is in white and has an unmodified T residue at the 3′-end of the primer strand; the structures are superimposed by the palm and fingers domains of the polymerase. Both, the AraC and the unmodified T sugar moieties have C2′-*endo* conformations.

In both complexes (Figs 3A and 4A), Polη embraces the template–primer with its palm (residues 1–13 and 90–238), fingers (residues 17–87), thumb (residues 241–301) domains and the PAD (polymerase associated domain or the little finger domain; residues 319–432) that is unique to Y-family polymerases. The palm domain provides the catalytic residues Asp13, Asp115, and Glu116, while the fingers domain is positioned above the templating base. The thumb and the PAD grip the template–primer DNA duplex from the minor and major groove surfaces, respectively (Figs 3A and 4A).

### Binary complex

AraC is covalently incorporated into the primer strand and its base makes a normal Watson-Crick base pair with a template G. The sugar of AraC stacks against the planar phenyl ring of Phe18 in the fingers domain (Fig. 3B,C). Phe18 occurs at a position in common with an aromatic residue in many other DNA polymerases, functioning as a “steric gate” for discrimination against the insertion of ribonucleotides (NTPs). That is, the orientation of 2′-OH on a NTP sugar is such that it would clash with the aromatic residue and the NTP would be excluded from incorporation. Because the 2′-OH on an AraC sugar points in an opposite direction to that on an NTP sugar, it does not generate any steric clashes and provides a basis by which Polη can incorporate AraCTP but not an NTP into a growing DNA chain. AraC is further stabilized in the Polη active site by a hydrogen bond between its 3′-OH and the backbone N atom of Phe17 (Fig. 3C**)**.

Interestingly, the conformation of AraC sugar is C3′-*endo* (Fig. 3C,D). This was a surprise because an A-form RNA-like C3′-*endo* sugar pucker is considered unfavorable for an arabinonucleoside^15–17^. However, the C3′-*endo* conformation is stabilized in the Polη active site by a hydrogen bond and stacking interactions with Arg61, a residue unique to Polη. In addition, there is a direct hydrogen bond between 2′-OH of the AraC sugar and the main chain carbonyl of Ile48. Together, these interactions facilitate and stabilize the C3′-*endo* pucker even though it is unfavorable for an arabinonucleoside. Overall, it further supports the idea that DNA polymerases in general mold DNA into an A-form-like conformation at the primer terminus^18–26^.

### Ternary complex

The ternary complex provides a basis for the addition of dNTP from an AraC primer terminus. As expected, 1) AraC has incorporated at the 3′ end of the primer strand, 2) the polymerase has translocated to the next templating position, and 3) the dATP nonreactive analog dAMPNPP has entered the active site (Fig. 4A–C). The dAMPNPP sugar is juxtaposed against the phenyl ring of Phe18, and two Mg^2+^ ions (A and B) complete the active site. Mg_A_^2+^ is ligated by the α-phosphate of dAMPNPP, the carboxylates of Asp115 (2.55 Ả) and Glu116 (2.22 Ả), and a water molecule (Fig. 4B**)**. Mg_B_^2+^ is coordinated in the basal octahedral plane by the dAMPNPP β- and γ-phosphates, the carboxylates of Asp13 (2.15 Ả) and Asp115 (2.08 Ả) and by the backbone carboxyl oxygen of Met14 (2.25 Ả). Thus, the overall configuration of the active site is very similar to that observed in previous Polη ternary complexes with unmodified DNAs and nonreactive dNTP analogs^27^, except that the 3′-OH of the AraC primer terminus is shifted by ~1.1 Å compared to the unmodified structures and is now too far away to interact with Mg_A_^2+^. This small shift of the 3′-OH is due to a slight tilt of the AraC sugar moiety (Fig. 3D). Hence, the χ torsion angle around the N-glycosidic bond that connects the base to the sugar (O4′-C1′-N1-C2) is changed from −115.0° in the unmodified 3′ primer T residue to −149.2° in the 3′ primer AraC. The altered position of the 3′-OH of AraC might underlie the slightly lower efficiency in Polη-catalyzed extension from AraC 3′-terminus relative to the unmodified primer base.

Compared to the binary complex, the AraC sugar at the primer terminus has switched to the normal for DNA B-form C2′-*endo* conformation (Figs 4B,C). It appears to be driven into this conformation by a direct hydrogen bond between 2′-OH of the AraC sugar and the non-bridging oxygen atom of the α-phosphate of dAMPNPP. Thus, in the C2′-*endo* conformation, the AraC sugar plays a direct role in binding of the incoming nucleotide. The 2′-OH of AraC sugar is also involved in a water-mediated hydrogen bond with Arg61. This has the effect of stabilizing Arg61 into a single rotameric conformation, as compared to multiple conformations of Arg61 in Polη ternary complex with unmodified primer terminus and nonreactive dNTP analogs (Fig. 4D)^27^.

## Discussion

Polη is currently the only known human DNA polymerase capable of efficiently extending DNA synthesis from an AraC–terminated primer. We show here that AraC is stabilized in the Polη active site via specific hydrogen bonding and stacking interactions. By contrast, human X-family polymerases Polλ and Polβ that function in base excision repair (BER) can insert AraC in a gapped DNA substrate, but there is no evidence to suggest that these polymerases can extend DNA synthesis from AraC-terminated primers^28,29^.

**Figure 5:**
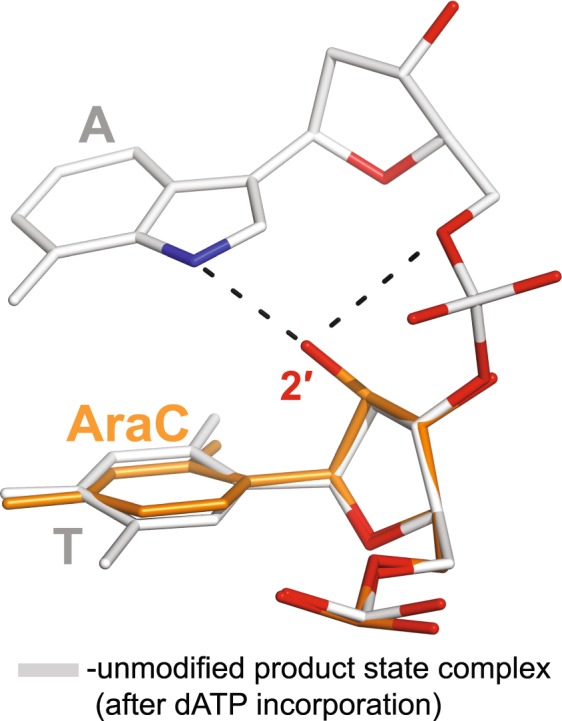
Model of the Polη AraC extension product complex. The C3′-*endo* AraC is superimposed onto the penultimate primer base in the product state of the unmodified complex after covalent insertion of an adenine base (PDB ID: 4ECX, 300 sec reaction time)^30^. The 2′-OH of the AraC makes hydrogen bonds with the O5′ of the phosphate group and with the N7 acceptor atom of the just incorporated adenine base.

Interestingly, a time-resolved crystallographic study of Polη’s catalytic cycle has revealed that the deoxyribose sugar of the 3′-terminal primer base changes its conformation from C2′-*endo* to C3′-*endo* during the phosphoryl transfer reaction^30^. This is apparently necessary to avoid clashes with the non-bridging oxygen of incoming nucleotide. We envisage a similar C2′-*endo* to C3′-*endo* transition of the AraC sugar during the catalytic reaction. Indeed, when we superimpose the C3′-*endo* AraC sugar (observed in our binary complex) onto the penultimate primer base in the time resolved structure (PDB ID: 4ECX, 300 sec reaction time)^30^ (Fig. 5), the “extra” 2′-OH on the AraC sugar is not only accommodated without any steric overlap, but can potentially make hydrogen bonds with the phosphate group and adenine base of the incorporated nucleotide (Fig. 5). Whether this creates sequence bias or mutagenic incorporation of purines during the extension of AraC remains to be determined.

Overall, the ability of the arabinose sugar in AraC to adopt or transition to the C3′-*endo* pucker is surprising. This is because the sugar in AraC (and other arabinonucleosides) is considered relatively restricted in conformation and preferentially adopts the C2′-*endo*^16^ or C1′-*exo*^17^ puckers. However, we show that this conformational restriction is easily surmounted in the Polη active site by interactions that 2′-OH of the C3′-*endo* sugar is able to establish with the polymerase. It would be interesting to know whether a more “rigid” sugar in AraC reduces the ability of Polη to extend DNA synthesis: lending to increased efficacy in the treatment of AML. In particular, locked conformation nucleotide analog chemistry may be helpful in the design and synthesis of such conformationally restricted AraC-like analogs^31^.

## Methods

### Preparation of protein and DNA for crystallization

An N-terminal His_6_ tagged catalytic core of human Polη (residues 1–432) with a C406M mutation was overexpressed in *Escherichia coli* and purified as previously described^27,32^. Briefly, the His_6_ tag was removed by overnight incubation with PreScission protease, and the protein was purified by ion-exchange (MonoS) chromatography followed by size-exclusion (Superdex 75). The protein was concentrated to ~1.3 mg/ml in 25 mM tris (pH 8.0), 250 mM NaCl, and 2 mM tris(2-carboxyethyl) phosphate (TCEP) and stored in aliquots at −80 °C.

### Crystallization

The crystals of the post-insertion AraC binary complex were obtained by incubating the human Polη catalytic core with a template-primer (5′-CATGACAGTGCT-3′/5′-AGCACTGT-3′) (Glen Research, Inc) in the presence of AraCTP (TriLink Biotechnologies inc.) by the hanging drop method against a reservoir solution containing 0.1 M MES pH 6.0 buffer and 10–14% PEG1500. Briefly, the template-primer DNAs were annealed and mixed with Polη in a 1.2:1 molar ratio to ~0.02 mM concentration of the complex in 25 mM tris (pH 8.0), 125 mM NaCl and 1 mM TCEP. The complex was concentrated with Amicon Ultra centrifugal filter (cut-off 3KDa) to a final complex concentration of ~0.105 mM. AraCTP and CaCl_2_ were then added to the complex to 2 mM and 4 mM concentrations, respectively. The complex was centrifuged at 8,000 rpm for 2 min at 4 °C. Crystallization drop was formed by mixing 1 μL of the complex with 1 μL of the reservoir solution and the crystals were grown at 20 °C. (The AraC residue was covalently added to the 3′ end of the primer strand under these conditions). A round of microseeding was necessary to produce larger diffraction-quality crystals. The crystals were cryoprotected in the reservoir solution supplemented with 24% PEG1500 and, sequentially, with 5%, 10% and 20% glycerol and flash frozen in liquid nitrogen for X-ray data collection.

To crystallize the extension ternary complex we prepared the binary polymerase complex with 12-nt template (5′-CATTGCAGTGCT-3′)/7-nt primer (5′-AGCACTG-3′) DNA as described above. We than added 2 mM AraCTP with 5 mM MgCl_2_ and incubated the reaction for 20 min at 16 °C. Following removal of the unincorporated AraCTP with Amicon Ultra centrifugal filter, we added a nonreactive dATP analog dAMPNPP (2′-deoxyadenosine-5′-[(α,β)-imido]triphosphate) at 2 mM and 5 mM MgCl_2_. The crystals were grown as described for the post-insertion binary complex.

### Structure determination and refinement

The X-ray diffraction data were collected at the NSLS X25 beam line at the Brookhaven National Laboratory and at the 24-ID NE-CAT beamline at Advanced Photon Source in Chicago. The data from the NSLS X25 beam line were processed and scaled using the HKL2000 suite^33^ and the data from the 24-ID NE-CAT beamline were processed by RAPD pipeline (http://necat.chem.cornell.edu/). We solved the structure by the molecular replacement method (Phaser)^34^ in the CCP4 program package^35^ using the Polη postinsertion binary complex structure (PDB ID: 4J9P) as a search model^36^. The model building, including substitution of the DNA sequence, was finished manually in Coot^37^ based on the electron density maps calculated in PHENIX Refine^38^. The final model was refined in PHENIX Refine^38^ and belongs to P6_1_ space group with unit cell dimensions of a = b = 98.6 Å, c = 81.5 Å, α = β = 90°, and γ = 120.0°. The structure has been refined to 1.83 Å resolution with R_free_ of 19.6% and R_work_ of 16.0% and consists of one Polη molecule (residues 1 to 432), one DNA template (residues 2 to 12), one DNA primer (residues 1 to 9), and a total of 436 solvent molecules. The placement of the AraC residue was verified using simulated annealing omit maps calculated in PHENIX^38^ with the AraC omitted from the model before heating to 2,000 K and then slowly cooling.

We solved the structure of the AraC-modified extension ternary complex by MR using the structure of Polη ternary complex (PDB ID 3MR2)^27^ as a search model. We refined the model as described above to 1.75 Å resolution in P6_1_ space group with unit cell dimensions of a = b = 98.8 Å, c = 81.8 Å, α = β = 90°, and γ = 120.0°; with R_free_ of 19.6% and R_work_ of 16.8%. The model consists of one Polη molecule (residues 1 to 432), one DNA template (residues 2 to 12), one DNA primer (residues 1 to 8), one dAMPNPP, two Mg^2+^ ions and a total of 383 solvent molecules.

The crystal data, together with the data collection and refinement statistics, are summarized in Table 1.

### Primer extension

We used two different DNA templates, G1 and G6, to examine extension from a C or AraC paired with an unmodified template **G**. The G1 template oligonucleotide contains the sequence 5′-GTCTAATACTTCCTAA**G**ATGCCTACACTGGAGTACCGGA-3′. The G6 template oligonucleotide contains a run of 6 G residues and it has the sequence 5′-GTCTAATACTTGGGGG**G**ATGCCTACACTGGAGTACCGGA-3′. We employed the 5′-^32^P labeled 23 nt primers 5′-TCCGGTACTCCAGTGTAGGCATX-3′ where X is a C or an AraC. Thus, DNA substrates for primer extension consisted of a ^32^P-radiolabeled oligonucleotide primer (23 nt) annealed to a 39 nt oligonucleotide DNA template by heating a mixture of primer/template at a 1:1.5 molar ratio to 95 °C and allowing it to cool to room temperature for several hours. The standard DNA polymerase reaction (5 μl) contained 25 mM tris (pH 7.5), 5 mM MgCl_2,_ 1 mM dithiothreitol, 100 μg/ml BSA, 10% glycerol, 10 nM DNA substrate, and 1 nM of Polη. Primer extension was assayed at 37 °C for 10 min in the presence of 25 μM of dATP, dGTP, and dTTP for G1 template and 50 μM each of dATP, dGTP, dTTP and dCTP (Roche Biochemicals, Indianapolis) for extension from C and 50 μM each of dATP, dGTP, dTTP and araCTP for extension from AraC on G6 template DNA. Reactions were stopped by the addition of loading buffer (95% formamide, 0.05% cyanol blue and 0.05% bromophenol blue) and the reaction products were than resolved on a 12% polyacrylamide gel containing 8 M urea. Gels were dried before autoradiography with a PhosphorImager.

## Electronic supplementary material

Supplementary Information

## Data Availability

Atomic coordinates and structure factors have been deposited deposited in the Protein Data Bank under accession codes 6D0M and 6D0Z for the AraC post-insertion binary and extension ternary complexes, respectively.

## References

[1] Magina KN (2017). Cytarabine dose in the consolidation treatment of AML: a systematic review and meta-analysis. Blood.

[2] Kadia TM, Ravandi F, O’Brien S, Cortes J, Kantarjian HM (2015). Progress in acute myeloid leukemia. Clin Lymphoma Myeloma Leuk.

[3] Lichtman MA (2013). A historical perspective on the development of the cytarabine (7days) and daunorubicin (3days) treatment regimen for acute myelogenous leukemia: 2013 the 40th anniversary of 7 + 3. Blood Cells Mol Dis.

[4] Cancer Facts and Figures 2017. *American Cancer Society* (2017).

[5] Grant S (1998). Ara-C: cellular and molecular pharmacology. Adv Cancer Res.

[6] Lujan SA, Williams JS, Kunkel TA (2016). DNA Polymerases Divide the Labor of Genome Replication. Trends Cell Biol.

[7] Jain R, Aggarwal AK, Rechkoblit O (2018). Eukaryotic DNA polymerases. Current Opinion in Structural Biology.

[8] Mikita T, Beardsley GP (1988). Functional consequences of the arabinosylcytosine structural lesion in DNA. Biochemistry.

[9] Perrino FW, Mekosh HL (1992). Incorporation of cytosine arabinoside monophosphate into DNA at internucleotide linkages by human DNA polymerase alpha. J Biol Chem.

[10] Perrino FW, Mazur DJ, Ward H, Harvey S (1999). Exonucleases and the incorporation of aranucleotides into DNA. Cell Biochem Biophys.

[11] Tsuda M (2017). The dominant role of proofreading exonuclease activity of replicative polymerase epsilon in cellular tolerance to cytarabine (Ara-C). Oncotarget.

[12] Major PP, Egan EM, Herrick DJ, Kufe DW (1982). Effect of ARA-C incorporation on deoxyribonucleic acid synthesis in cells. Biochem Pharmacol.

[13] Prakash S, Johnson RE, Prakash L (2005). Eukaryotic translesion synthesis DNA polymerases: specificity of structure and function. Annu Rev Biochem.

[14] Chen YW, Cleaver JE, Hanaoka F, Chang CF, Chou KM (2006). A novel role of DNA polymerase eta in modulating cellular sensitivity to chemotherapeutic agents. Mol Cancer Res.

[15] Teng MK, Liaw YC, van der Marel GA, van Boom JH, Wang AH (1989). Effects of the O2′ hydroxyl group on Z-DNA conformation: structure of Z-RNA and (araC)-[Z-DNA]. Biochemistry.

[16] Gmeiner WH, Konerding D, James TL (1999). Effect of cytarabine on the NMR structure of a model okazaki fragment from the SV40 genome. Biochemistry.

[17] Li F (2006). 2′-Fluoroarabino- and arabinonucleic acid show different conformations, resulting in deviating RNA affinities and processing of their heteroduplexes with RNA by RNase H. Biochemistry.

[18] Doublie S, Tabor S, Long AM, Richardson CC, Ellenberger T (1998). Crystal structure of a bacteriophage T7 DNA replication complex at 2.2 A resolution. Nature.

[19] Li Y, Korolev S, Waksman G (1998). Crystal structures of open and closed forms of binary and ternary complexes of the large fragment of Thermus aquaticus DNA polymerase I: structural basis for nucleotide incorporation. EMBO J.

[20] Franklin MC, Wang J, Steitz TA (2001). Structure of the replicating complex of a pol alpha family DNA polymerase. Cell.

[21] Johnson SJ, Taylor JS, Beese LS (2003). Processive DNA synthesis observed in a polymerase crystal suggests a mechanism for the prevention of frameshift mutations. Proc Natl Acad Sci USA.

[22] Batra VK (2006). Magnesium-induced assembly of a complete DNA polymerase catalytic complex. Structure.

[23] Berman AJ (2007). Structures of phi29 DNA polymerase complexed with substrate: the mechanism of translocation in B-family polymerases. EMBO J.

[24] Garcia-Diaz M, Bebenek K, Krahn JM, Pedersen LC, Kunkel TA (2007). Role of the catalytic metal during polymerization by DNA polymerase lambda. DNA Repair (Amst).

[25] Wang F, Yang W (2009). Structural Insight into Translesion Synthesis by DNA Pol II. Cell.

[26] Swan MK, Johnson RE, Prakash L, Prakash S, Aggarwal AK (2009). Structural basis of high-fidelity DNA synthesis by yeast DNA polymerase delta. Nat Struct Mol Biol.

[27] Biertumpfel C (2010). Structure and mechanism of human DNA polymerase eta. Nature.

[28] Chrencik JE, Burgin AB, Pommier Y, Stewart L, Redinbo MR (2003). Structural impact of the leukemia drug 1-beta-D-arabinofuranosylcytosine (Ara-C) on the covalent human topoisomerase I-DNA complex. J Biol Chem.

[29] Prakasha Gowda AS, Polizzi JM, Eckert KA, Spratt TE (2010). Incorporation of gemcitabine and cytarabine into DNA by DNA polymerase beta and ligase III/XRCC1. Biochemistry.

[30] Nakamura T, Zhao Y, Yamagata Y, Hua YJ, Yang W (2012). Watching DNA polymerase eta make a phosphodiester bond. Nature.

[31] Singh, S. K., Nielsen, P., Koshkin, A. A. & Wengel, J. LNA (locked nucleic acids): synthesis and high-affinity nucleic acid recognition. *Chem Commun*, 455-456, 10.1039/a708608c (1998).

[32] Ummat A (2012). Structural basis for cisplatin DNA damage tolerance by human polymerase eta during cancer chemotherapy. Nat Struct Mol Biol.

[33] Otwinowski Z, Minor W (1997). Processing of X-ray diffraction data collected in oscillation mode. Methods Enzymol.

[34] McCoy AJ (2007). Phaser crystallographic software. J Appl Crystallogr.

[35] Winn MD (2011). Overview of the CCP4 suite and current developments. Acta Crystallogr D Biol Crystallogr.

[36] Zhao Y (2013). Mechanism of somatic hypermutation at the WA motif by human DNA polymerase eta. Proc Natl Acad Sci USA.

[37] Emsley P, Cowtan K (2004). Coot: model-building tools for molecular graphics. Acta Crystallogr D Biol Crystallogr.

[38] Adams PD (2010). PHENIX: a comprehensive Python-based system for macromolecular structure solution. Acta Crystallogr D Biol Crystallogr.

