# Supplementary material for: Structural basis for polymerase η–promoted resistance to the anticancer nucleoside analog cytarabine
Source: Sci Rep. 2018 Aug 23;8:12702. doi: 10.1038/s41598-018-30796-w (PMC6107656; doi:10.1038/s41598-018-30796-w)
Supplement: Supplementary file 1 — Supplementary Information [file 41598_2018_30796_MOESM1_ESM.pdf]

SUPPLEMENTARY INFORMATION

Structural basis for polymerase η–promoted resistance to the anticancer nucleoside analog cytarabine

Olga Rechkoblit, Jayati Roy Choudhury, Angeliki Buku, Louise Prakash, Satya Prakash, and Aneel K. Aggarwal

Supplementary Figures 1 and 2

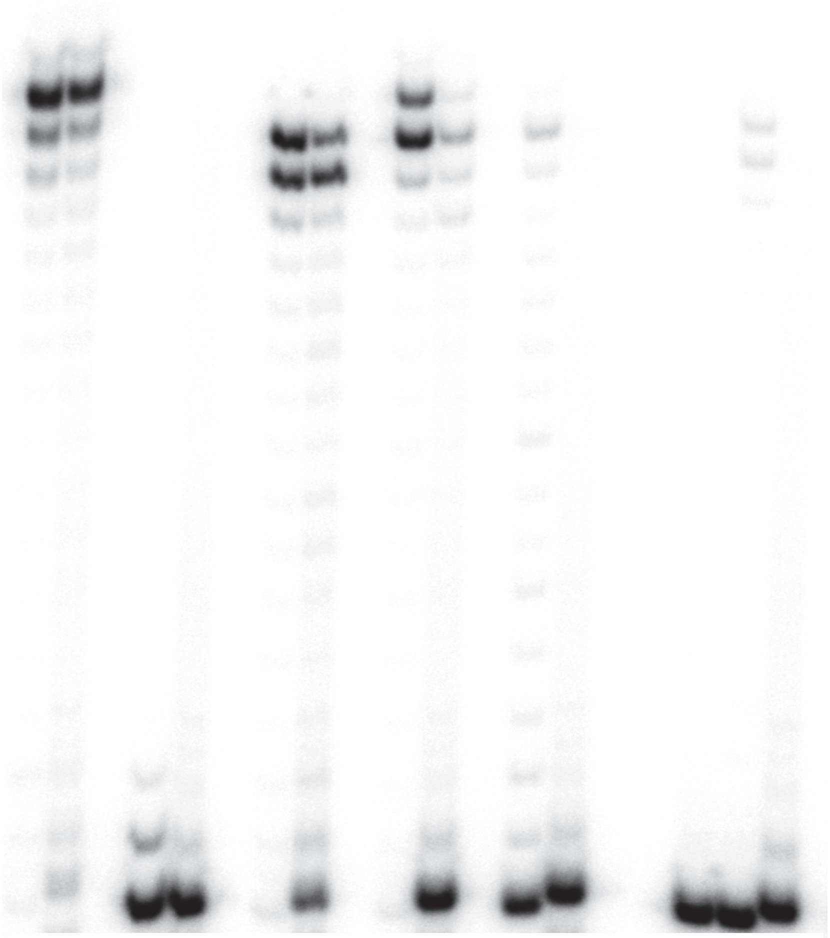

Lane no: 2 3 1

Supplementary Figure 1

Supplementary Figure 1. The original gel related to Fig. 2A.

Extension of ^{32}P 5′-endlabeled 23-nt primer with the unmodified C or AraC residues at the 3′-end bound to the 5′-…CCTAA**G**A…-3′ 39-nt template in the presence of dATP, dGTP, and dTTP. Lane 1, unmodified DNA substrate; Lane 2, extension from the unmodified C; Lane 3, extension from AraC. To produce Fig. 2A, lane 1 and lanes 2 and 3 were cropped from different parts of the gel and aligned together. Thus, in Fig. 2A, lane 1 is separated from lanes 2 and 3 with white space. All extension reactions were conducted at 37°C for 10 min.

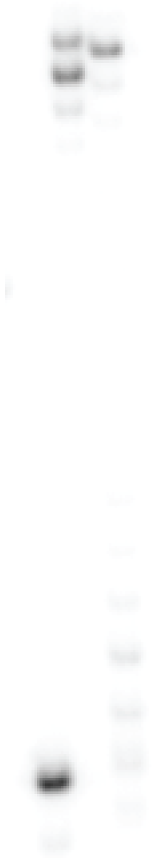

Lane no: 1 2 3

Supplementary Figure 2

Supplementary Figure 2. The original gel related to Fig. 2B.

Extension with the 5′-…GGGGG**G**A…-3′ 39-nt template. Lane 1, unmodified DNA substrate; Lane 2, extension from the unmodified C in the presence of dATP, dGTP, dTTP and dCTP; Lane 3, extension from the AraC in the presence of dATP, dGTP, dTTP and AraCTP. All extension reactions were conducted at 37°C for 10 min.
